# Comparative Effectiveness of Rituximab and Common Induction Therapies for Lupus Nephritis: A Systematic Review and Network Meta-Analysis

**DOI:** 10.3389/fimmu.2022.859380

**Published:** 2022-04-04

**Authors:** Kang Li, Yanqiu Yu, Yuan Gao, Fei Zhao, Zheng Liang, Junjie Gao

**Affiliations:** Department of Nephrology, Cangzhou Central Hospital, Cangzhou, China

**Keywords:** lupus nephritis, rituximab, tacrolimus, mycophenolate mofetil, Bayesian network meta-analysis

## Abstract

**Objective:**

This study aimed to compare the efficacy and safety (infection events) between rituximab (RTX), tacrolimus (TAC), mycophenolate mofetil (MMF), and cyclophosphamide (CYC) as induction therapies in lupus nephritis (LN).

**Methods:**

Electronic databases, including PubMed, EMBASE, and the Cochrane Library, were searched from inception up to December 9, 2021. Bayesian network meta-analysis was used to combine the direct and indirect evidence of different drugs for LN patients. The pooled relative effects were shown using odds ratios (ORs) and 95% credible intervals (CrIs).

**Results:**

Nineteen studies (1,566 patients) met the inclusion criteria and were selected in the present study. The network meta-analysis reported that no statistically significant differences were found in partial remission (PR) and infection among the four drugs. RTX showed a significantly higher complete remission (CR) than MMF (OR = 2.60, 95% CrI = 1.00–7.10) and seemed to be more effective than CYC (OR = 4.20, 95% CrI = 1.70–14.00). MMF had a better CR than CYC (OR = 1.60, 95% CrI = 1.00–3.20). TAC presented a better overall response than CYC (OR = 3.70, 95% CrI = 1.20–12.00). Regarding CR and overall response, the maximum surface under the cumulative ranking curve (SUCRA) values were 96.94% for RTX and 80.15% for TAC. The maximum SUCRA value of infection reaction was 74.98% for RTX and the minimum value was 30.17% for TAC, respectively.

**Conclusions:**

RTX and TAC were the most effective drugs for induction remission in LN. Among the four drugs, TAC had the lowest probability of infection, and RTX showed the highest probability of experiencing an infection. This meta-analysis could not conclude about other adverse events.

## Introduction

Lupus nephritis (LN) is one of the most common clinical manifestations and serious complications of systemic lupus erythematosus (SLE) and is a leading cause of morbidity and mortality in SLE (1–3). About 40% of patients with lupus develop LN (2). The management of LN comprises two phases. The induction phase aims to induce remission. The maintenance phase aims to prevent relapse and progression to end-stage renal disease. Glucocorticoids are the first treatment and improve the renal outcomes of LN. Then, they are followed by cyclophosphamide (CYC), mycophenolate mofetil (MMF), and tacrolimus (TAC) (4, 5). CYC regimens have long been considered the gold standard for inducing renal remission and preventing relapse (4). Increasing evidence shows that MMF and TAC are at least equivalent to CYC for induction and maintenance treatment of severe LN (6–8). Nevertheless, the adverse effects of these drugs can be considerable and limiting. For example, the benefits of CYC are outweighed by treatment-related adverse effects, including gonadal toxicity (9). Although the renal remission rate with CYC or MMF regimens is up to 50%–80% in LN patients, many of these responses are partial (10). Therefore, it is urgent to search for better treatments that have better efficacy and fewer side effects (including sparing fertility) to manage LN.

Rituximab (RTX) is a chimeric monoclonal antibody that targets CD20. RTX offers an alternative or adjunctive option for SLE patients (11–13). Recently, RTX has been introduced as an induction drug for LN, and studies suggested that RTX seemed to be at least as effective as MMF and CYC regimens in inducing remission (14, 15). Still, few studies were conducted to compare the efficacy and safety of RTX with common therapeutic drugs, especially TAC. Therefore, it is important for medical decisions to assess the relative value between intervention and comparators. Network meta-analysis is an extension of traditional pairwise meta-analysis and simultaneously combines direct and indirect information about the relative efficacy of each treatment (16). Thus, using a network meta-analysis, the present systematic study aimed to compare the relative efficacy and safety (infection events) of RTX, TAC, and MMF as LN induction therapy.

## Methods

### Search Strategy

The present meta-analysis was reported according to the Preferred Reporting Items for Systematic Reviews and Meta-analyses (PRISMA) network meta-analysis extension statement (17, 18). Electronic databases, including PubMed, EMBASE, and the Cochrane Library, were systematically searched from inception to December 9, 2021. The search terms consisted of “lupus nephritis”, “LN”, “systemic lupus erythematosus”, “SLE”, “rituximab”, “RTX”, “mycophenolate mofetil”, “MMF”, “tacrolimus” and “TAC”. The reference lists from potential studies were also manually searched.

### Inclusion and Exclusion Criteria

According to the Population, Intervention, Comparison, Outcomes, and Study designs (PICOS) structure, the inclusion criteria were 1) patients with LN (population); 2) studies that examined the efficacy or safety of RTX, TAC, MMF, or CYC (intervention and comparator); 3) available data, effectiveness, or adverse effects (outcome); and 4) observational study or randomized controlled trials (RCTs) (study design). The present study excluded all letters, comments, case reports, animal models, and reviews.

### Data Extraction and Quality Assessment

Two authors independently collected the data, including the first author’s surname, the year of publication, country, study design, sample size, the ratio of male/female, kidney biopsy class, duration of follow-up, and treatment drugs (intervention and comparators). In case of disagreement, group discussion was used to resolve any discrepancy. The efficacy indicators were the number of patients who achieved complete remission (CR) and partial remission (PR). CR and PR definitions were based on the remission criteria used in each trial. The overall response was determined as the number of cases with CR+PR. The safety indicator was the number of patients that suffered from infection, including upper respiratory infection, sepsis, or pneumonia. The Newcastle-Ottawa Scale (NOS) (19) was adopted to assess the methodological quality of observational studies in our meta-analysis. The score ranged from 0 to 9, with a total score of ≥7 indicating high quality and <7 indicating low quality. The Jadad score (20) was used to evaluate the quality of RCTs; it ranges from 0 to 7 stars, with a total score ≥4 as high quality and <4 as low quality.

### Statistical Analysis

Stata (Version 14.0) and R (Version 4.1.1) were used in the present study. In network analysis, the Bayesian random-effects model was performed to combine both direct and indirect information for therapeutic drugs of LN, combining the information efficacy and safety indicators from different studies. The Bayesian Markov chain Monte Carlo method (21) was used to compute Bayesian consistency models using four chains with over-dispersed initial values with Gibbs sampling based on 20,000 iterations after a burn-in phase of 5,000 iterations. The pooling effect sizes were shown along with odds ratios (ORs) and 95% credible intervals (CrIs). The efficacy and safety of each treatment were compared using the surface under the cumulative ranking curve (SUCRA) (22), and the optimal treatment regimen was determined. SUCRA would be 1 when a treatment is certain to be the best and 0 when a treatment is certain to be the worst. Inconsistency refers to the extent of disagreement between direct and indirect evidence. Inconsistency assessment is an essential part of a network meta-analysis. The extent of disagreement between direct and indirect comparison results in NMA was evaluated using the node splitting analysis. If *p* > 0.05, there was consistency between the direct and indirect comparison results. Otherwise, it was inconsistent. Publication bias was assessed using funnel plots.

## Results

### Included Study Characteristics

**Figure 1:**
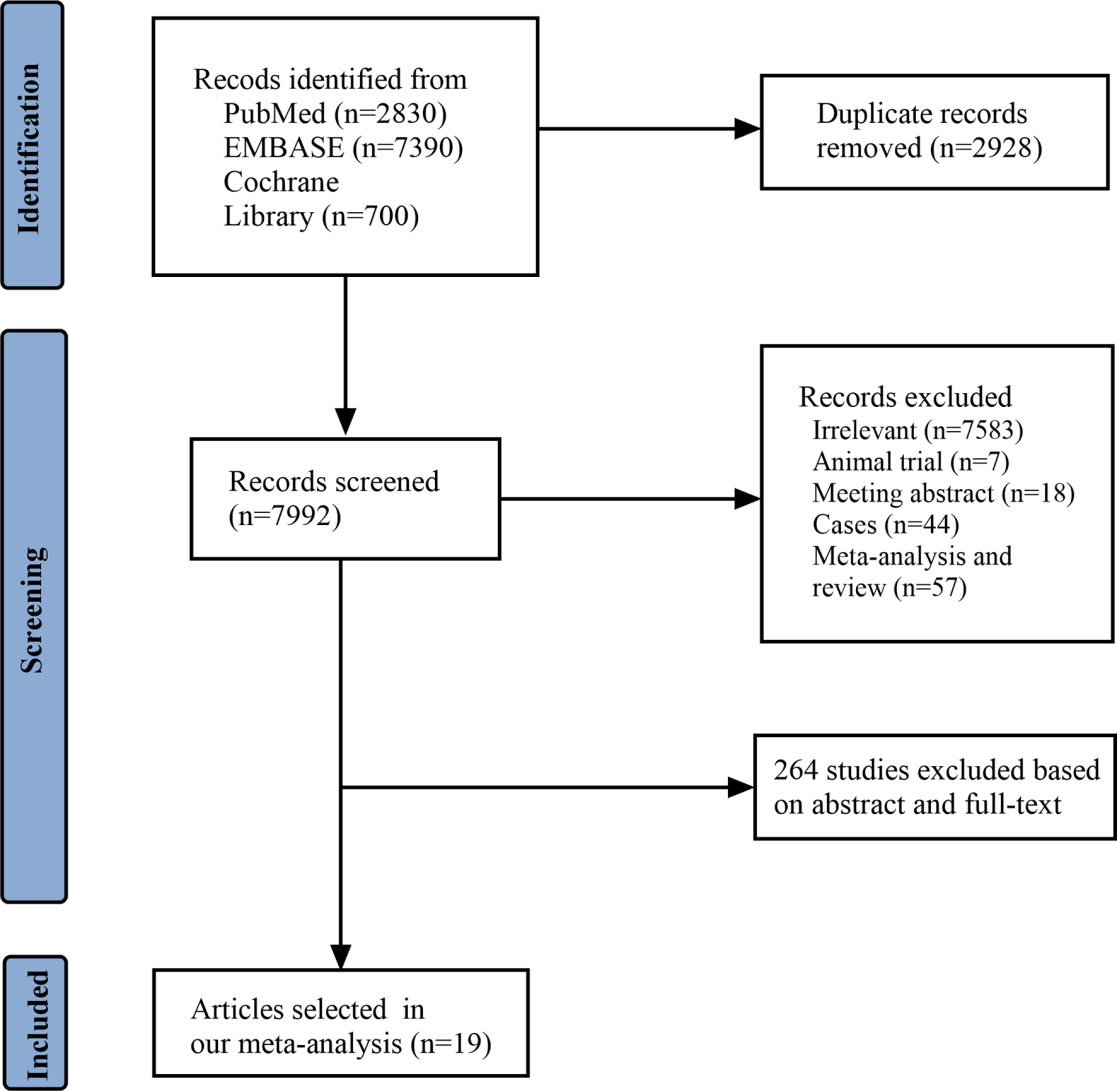
Flow diagram of study identification and selection.

**Figure 2:**
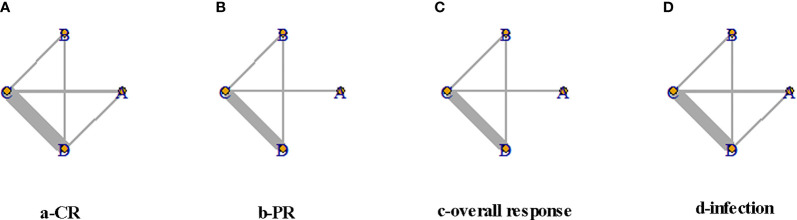
Network diagram for complete remission (CR), partial remission (PR), overall response, and infection events. The size of each node is proportional to the sample size of the individual treatment regimen; the widths of the connecting lines are proportional to the number of studies compared between the two regimens. **(A)** Rituximab (RTX). **(B)** Tacrolimus (TAC). **(C)** Mycophenolate mofetil (MMF). **(D)** Cyclophosphamide (CYC). CR, complete remission; PR, partial remission.

A total of 10,920 articles were initially selected. Among them, 19 studies met inclusion. Therefore, 19 articles (6–8, 14, 23–36) involving 1,566 LN patients were included in the present study. Among the 19 studies, 14 were from Asian countries (Pakistan, China, India, Nepal, Japan, Korea, and Malaysia), one was from Egypt, and three were from Western countries (UK, USA, and Italy). Two studies were three-arm studies, and the others were two-arm studies. **Table 1** reports the basic characteristics of the included studies. Among them, 12 studies were RCTs, and seven were observational studies. Only eight studies were considered high-quality studies according to the NOS or Jadad score (**Tables 2**, **3**). **Figure 1** illustrates the stages in selecting studies for inclusion in the study. **Figure 2** presents the network diagram of evidence for treatment efficacy and safety of each regimen.

**Table 1:** Characteristics of included studies in the network meta-analysis.

Study	Study design	Sample size	Ratio of M/F	Biopsy class	Follow-up	Treatments drugs	Study quality
Gul et al. (6), Pakistan	RCT	28	All female	III, IV, V	24 weeks	MMF vs. CYC	3
Appel et al. (23), USA	RCT	370	1:1.5	III, IV, V	24 weeks	MMF vs. CYC	3
Wang et al. (7), China	PC	40	1:4.0	III, IV, V	12 months	TAC vs. CYC	7
Yap et al. (24), China	RCT	16	1:1.7	V	24 months	MMF vs. TAC	3
Goswami et al. (25), India	CT	83	1:17.1	III, IV, V	6 months	MMF vs. RTX	7
Basu et al. (14), India	RC	42	1:1.1	IV, V	36 months	RTX vs. MMF vs. CYC	4
Sundel et al. (26), UK	RCT	346	1:5.7	III, IV, V	24 weeks	MMF vs. CYC	6
Wang et al. (28), China	RCT	20	NA	IV	6 months	MMF vs. CYC	3
Lau et al. (29), USA	RT	13	1:12	III	6 months	MMF vs. CYC	5
El-Shafey et al. (30), Egypt	RCT	47	1:22.5	III, IV	24 weeks	MMF vs. CYC	3
Sedhain et al. (31), Nepal	RCT	42	1:7.4	III, IV, V	24 weeks	MMF vs. CYC	3
Feng et al. (32), China	RCT	53	1:7.6	III, IV, V	24 weeks	MMF vs. CYC	5
Mendonca et al. (33), India	RCT	37	1:4	III, IV, V	24 weeks	MMF vs. CYC	3
Onishi et al. (27), Japan	RT	21	All female	III, IV, V	24 weeks+	MMF vs. CYC	5
Joo et al. (34), Korea	CT	99	1:10	III, IV, V	12 months	MMF vs. CYC	7
Ong et al. (35), Malaysia	RCT	44	1:5.3	III, IV, V	6 months	MMF vs. CYC	5
Moroni et al. (15), Italy	CT	34	1:7.5	III, IV, V	12 months	RTX vs. MMF vs. CYC	5
Chen et al. (8), China	RCT	81	1:5.8	III, IV, V	6 months	TAC vs. CYC	5
Mok et al. (36), Hong Kong	RCT	150	1:11.5	V	6 months	MMF vs. TAC	5

CT, controlled trial; CYC, cyclophosphamide; M/F, male/female; MMF, mycophenolate mofetil; NA, not available; PC, prospective cohort; RC, retrospective cohort; RCT, randomized controlled trial; RTX, rituximab; TAC, tacrolimus.

**Table 2:** Study quality—Newcastle-Ottawa Scale (NOS) for observational studies.

Study	1	2	3	4	5	6	7	8	Total
Wang et al.	1	1	1	1	1	1	0	1	7
Goswami et al.	1	1	1	1	1	1	0	1	7
Basu et al.	0	0	1	1	0	1	0	1	4
Lau et al.	0	0	1	1	1	1	0	1	5
Onishi et al.	0	0	1	1	1	1	0	1	5
Joo et al.	1	1	1	1	1	1	0	1	7
Moroni et al.	0	0	1	1	1	1	0	1	5

1, representativeness of the exposed cohort; 2, selection of the non-exposed cohort; 3, ascertainment of exposure; 4, demonstration that outcome of interest was not present at the start of the study; 5, comparability of cohorts on the basis of the design analysis; 6, assessment of outcomes; 7, follow-up was long enough for outcomes to occur; 8, adequacy of follow-up of cohorts.

**Table 3:** Study quality—Jadad score for RCTs.

Study	1	2	3	4	Total
Gul et al.	1	1	0	1	3
Appel et al.	1	1	0	1	3
Yap et al.	1	1	0	1	3
Sundel et al.	2	2	1	1	6
Wang et al.	1	1	0	1	3
El-Shafey et al.	1	1	0	1	3
Sedhain et al.	1	1	0	1	3
Feng et al.	2	2	0	1	5
Mendonca et al.	1	1	0	1	3
Ong et al.	2	2	0	1	5
Chen et al.	2	2	0	1	5
Mok et al.	2	2	0	1	5

1, randomization; 2, concealment of allocation; 3, double blinding; 4, withdrawals and dropouts; RCTs, randomized controlled trials.

### Analysis of Inconsistency and Detection of Publication Bias

The node splitting analysis was used to evaluate network inconsistency between direct and indirect comparison results and suggested no inconsistency (*p* > 0.05) (**Supplementary Figures 1** **–** **4**). The publication bias was assessed using a funnel plot. In this analysis, the funnel plot was asymmetric, suggesting the presence of publication bias in the direct comparison meta-analysis (**Supplementary Figures 5** **–** **8**).

### Comparison of Efficacy of Treatment Regimen

**Figure 3:**
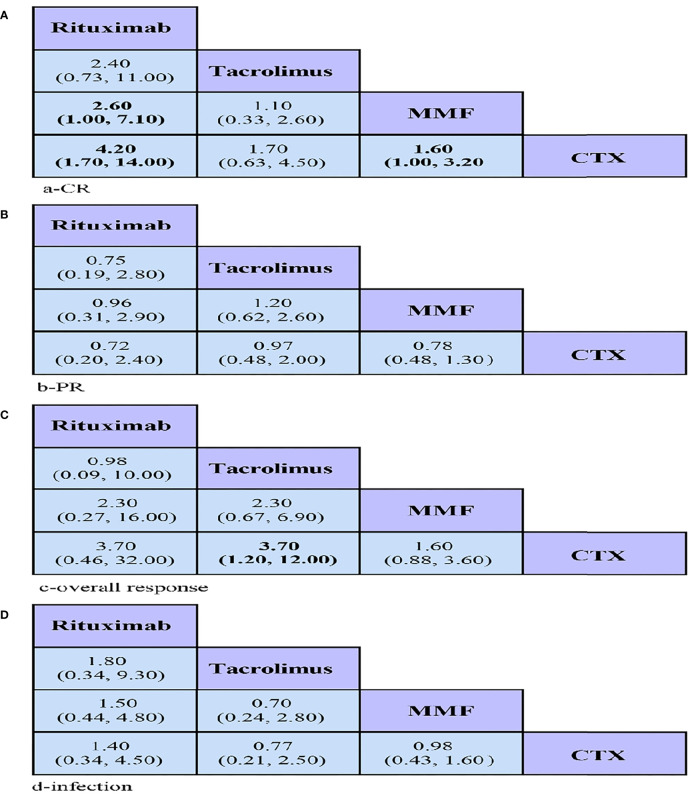
League tables showing the results of comparing the efficacy and safety of all drugs, including odds ratios (OR) and 95% credible intervals in the network meta-analyses. **(A–C)** Efficacy: OR > 1 means the drug in the top left is better. **(D)** Safety: OR < 1 means the treatment in the top left is better. CR, complete remission; PR, partial remission; MMF, mycophenolate mofetil; CYC, cyclophosphamide.

In the present study, the numbers of CR, PR, and overall responses were considered as efficacy outcomes. In terms of CR, RTX showed a significantly higher CR than MMF (OR = 2.60, 95% CrI = 1.00–7.10) and seemed to be more effective than CYC (OR = 4.20, 95% CrI = 1.70–14.00) (**Figure 3A**). Similarly, MMF had a better CR than CYC (OR = 1.60, 95% CrI = 1.00–3.20) (**Figure 3C**). Regarding overall response, TAC presented a better overall response than CYC (OR = 3.70, 95% CrI = 1.20–12.00). As for PR, no significant differences were found among the four drugs (**Figure 3B**).

### Comparison of Safety of Treatment Regimen

Comparison of infection suggested no statistically significant differences among any of the groups (**Figure 3D**).

### Result Sorting

**Table 4** presents the SUCRA values (%) for treatment efficacy and safety of each regimen. Regarding CR, the maximum SUCRA value of RTX was 96.94%, suggesting that RTX was likely to achieve the highest CR among these four treatment drugs. In terms of overall response, the maximum SUCRA value of TAC was 80.15%, meaning that TAC had the highest overall response among these four drugs. Besides, CYC was likely to achieve the highest PR among drugs, as it presented a higher probability of PR (SUCRA = 69.47%). As for safety, ranking probability based on SUCRA suggested that TAC was the safest treatment, as its minimum SUCRA value was 30.17%.

**Table 4:** Rank probability of efficacy and safety for each treatment drug.

Treatments	Complete remission	Partial remission	Total remission	Infection
RTX	96.94	35.29	73.57	74.98
TAC	49.86	62.72	80.15	30.17
MMF	48.34	32.53	40.47	45.71
CYC	4.87	69.47	5.81	49.14

CYC, cyclophosphamide; RCT, randomized controlled trial; RTX, rituximab; TAC, tacrolimus.

## Discussion

It is important for induction of remission to achieve the best long-term outcomes among patients with LN. The present network meta-analysis combined and compared available evidence of the effectiveness and safety (infection events) of RTX, TAC, MMF, and CYC based on the number of patients who achieved CR, PR, and overall response and who suffered from infection events. Network meta-analysis can simultaneously combine direct and indirect information of the relative efficacy and safety of each treatment to determine the optimal treatment regimen even if there are no or insufficient head-to-head comparisons (37, 38).

In the present study, RTX was the most effective drug for inducing CR among LN patients, followed by TAC and MMF. CYC was the most successful medicine for inducing PR among patients with LN, followed by TAC and RTX. In terms of overall response, TAC was the most effective treatment drug for achieving overall response among LN patients, followed by RTX and MMF. As for safety, TAC was the safest treatment with the lowest likelihood of infection events. However, RTX showed the highest probability of experiencing infection without considering other adverse events.

In summary, RTX and TAC were the most effective drugs for inducing remission among LN patients, and TAC had the lowest probability of infection compared with the other drugs. These results were consistent with a previous network meta-analysis that compared the efficacy and safety of TAC, MMF, and CYC regimens (39). Lee and Song found that TAC was the most effective induction treatment for patients with LN and had the highest probability of decreasing the risk of serious infections (39). In addition, our results were in accordance with another meta-analysis in which the direct comparison reported that TAC was more effective and safer than CYC (40). A review showed that LN patients would benefit from RTX (41). A prior study found that RTX presented a satisfying efficacy and safety for SLE patients (42). Besides, RTX therapy has been used in vasculitis and rheumatoid arthritis. Although RTX is widely used in patients with LN, RTX as an induction treatment is less established than MMF and CYC. A possible reason is the lack of large-scale placebo-controlled clinical trials (41). In addition, the evidence for RTX use remains varied in terms of ethnicity. For example, an RCT comparing the efficacy of RTX with placebo among patients with extrarenal diseases found that there was no significant difference in disease response rate between the two groups (43). However, the RTX treatment group in a subgroup analysis showed a significantly higher renal response rate among Hispanic and African-American patients. In the present study, RTX showed the highest risk of experiencing infection adverse events. A study found that infection, infusion reaction, and neutropenia were the most common adverse events of RTX (44). Furthermore, with respect to infection, evidence reported that a dose-dependent relationship was found between the RTX dose and the frequency of infection (41). Besides, RTX, as induction therapy for LN, is mainly used in the Americas (USA, Mexico, etc.), Europe (the United Kingdom, Italy, Sweden, Spain, Greece, Netherlands, etc.), Asia (Japan, China, Singapore, etc.), and Australia. TAC is only used in Asian countries, and the use of TAC is not recommended by the European League Against Rheumatism (EULAR). The off-label use of RTX in SLE was first reported in 2002 and has been increasingly used in patients with SLE (45). Therefore, off-label use should be carefully considered.

Some limitations might be considered in this network meta-analysis. First, in addition to RCTs, observational studies were included, a problem particularly found in RTX since there were no RCTs of RTX. Besides, some RCTs included in our study had small sizes. Hence, more RCTs with a large sample size should be conducted to compare RTX, TAC, MMF, and CYC, especially head-to-head trials. Second, the data and the methods of reporting data in the primary trials limited the results in our study due to different definitions in each study. For instance, the CR and PR results were heterogeneous owing to variable definitions in existing studies. Still, our results found a low heterogeneity in the pooled analysis, which suggested that our network meta-analysis was appropriate. Furthermore, heterogeneity in patients’ characteristics of eligible studies in our meta-analysis affected the results of our study. For example, Mohan et al. reported that the response to treatment drugs for LN patients varied with ethnicity (46). In addition, Merrill et al. found that the RTX treatment group showed a significantly higher renal response rate between Hispanic and African-American patients (43). Besides, prior evidence found that diet and exercise influenced renal function (47, 48). However, these factors were not adjusted in the current meta-analysis owing to insufficient data in our study, which might have a potential effect on our results. Third, for some drugs, the data were limited in some direct comparisons. For example, in our study, direct comparison between RTX and TAC was insufficient, so indirect comparison among the two groups was limited. Fourth, with respect to adverse events, only infection events were considered in the present study. However, other adverse events, including gastrointestinal reaction, myelosuppression, liver damage, alopecia, leukopenia, and menstrual disorders, were not considered owing to insufficient data. Therefore, our results should be interpreted with caution.

Although these limitations have a potential impact on our results, our study had several strengths. First, the present study used a comprehensive literature search without date and language restriction. Second, though the sample sizes from individual trials ranged from 13 to 346, the pooled analysis in our study involved 1,566 LN patients. Finally, our study used a standardized analysis method to evaluate the confidence that might be held in our results. Network meta-analysis simultaneously combined direct and indirect information of the relative efficacy of each treatment even if there were a lack of direct head-to-head comparisons.

## Conclusion

In conclusion, our network meta-analysis combined and compared available evidence of the effectiveness and safety of RTX, TAC, MMF, and CYC based on the number of patients who achieved CR, PR, and overall response and those who suffered from infection events. RTX and TAC were the most effective drugs for induction remission among LN patients. TAC had the lowest probability of infection compared with other drugs. RTX showed the highest probability of experiencing an infection. This meta-analysis could not conclude about other adverse events.

## Data Availability Statement

The original contributions presented in the study are included in the article/**Supplementary Material**. Further inquiries can be directed to the corresponding author.

## Author Contributions

KL and JG carried out the studies, participated in collecting data, and drafted the manuscript. KL and YG performed the statistical analysis and participated in its design. KL, FZ, and ZL participated in the acquisition, analysis, or interpretation of data and drafted the manuscript. All authors read and approved the final manuscript.

## Conflict of Interest

The authors declare that the research was conducted in the absence of any commercial or financial relationships that could be construed as a potential conflict of interest.

## Publisher’s Note

All claims expressed in this article are solely those of the authors and do not necessarily represent those of their affiliated organizations, or those of the publisher, the editors and the reviewers. Any product that may be evaluated in this article, or claim that may be made by its manufacturer, is not guaranteed or endorsed by the publisher.

## Supplementary Material

Supplementary Figure 1 Network node-splitting analysis for complete remission. **(A)** Rituximab (RTX); **(B)** Tacrolimus (TAC); **(C)** Mycophenolate mofetil (MMF); **(D)** Cyclophosphamide (CYC); 95%CrI, 95% credibility interval.

Supplementary Figure 2 Network node-splitting analysis for partial remission. **(B)** Tacrolimus (TAC); **(C)** Mycophenolate mofetil (MMF); **(D)** Cyclophosphamide (CYC); 95%CrI, 95% credibility interval.

Supplementary Figure 3 Network node-splitting analysis for overall response. **(B)** Tacrolimus (TAC); **(C)** Mycophenolate mofetil (MMF); **(D)** Cyclophosphamide (CYC); 95%CrI, 95% credibility interval.

Supplementary Figure 4 Network node-splitting analysis for infection. **(A)** Rituximab (RTX); **(B)** Tacrolimus (TAC); **(C)** Mycophenolate mofetil (MMF); **(D)** Cyclophosphamide (CYC); 95%CrI, 95% credibility interval.

Supplementary Figure 5 Funnel plot for complete remission. RTX, Rituximab; TAC, Tacrolimus; MMF, Mycophenolate mofetil; CYC, Cyclophosphamide.

Supplementary Figure 6 Funnel plot for partial remission. RTX, Rituximab; TAC, Tacrolimus; MMF, Mycophenolate mofetil; CYC, Cyclophosphamide.

Supplementary Figure 7 Funnel plot for overall response. RTX, Rituximab; TAC, Tacrolimus; MMF, Mycophenolate mofetil; CYC, Cyclophosphamide.

Supplementary Figure 8 Funnel plot for infection. RTX, Rituximab; TAC, Tacrolimus; MMF, Mycophenolate mofetil; CYC, Cyclophosphamide.
