# Supplementary figures and images for: Comparative Effectiveness of Rituximab and Common Induction Therapies for Lupus Nephritis: A Systematic Review and Network Meta-Analysis
Source: Front Immunol. 2022 Apr 4;13:859380. doi: 10.3389/fimmu.2022.859380 (PMC9013779; doi:10.3389/fimmu.2022.859380)

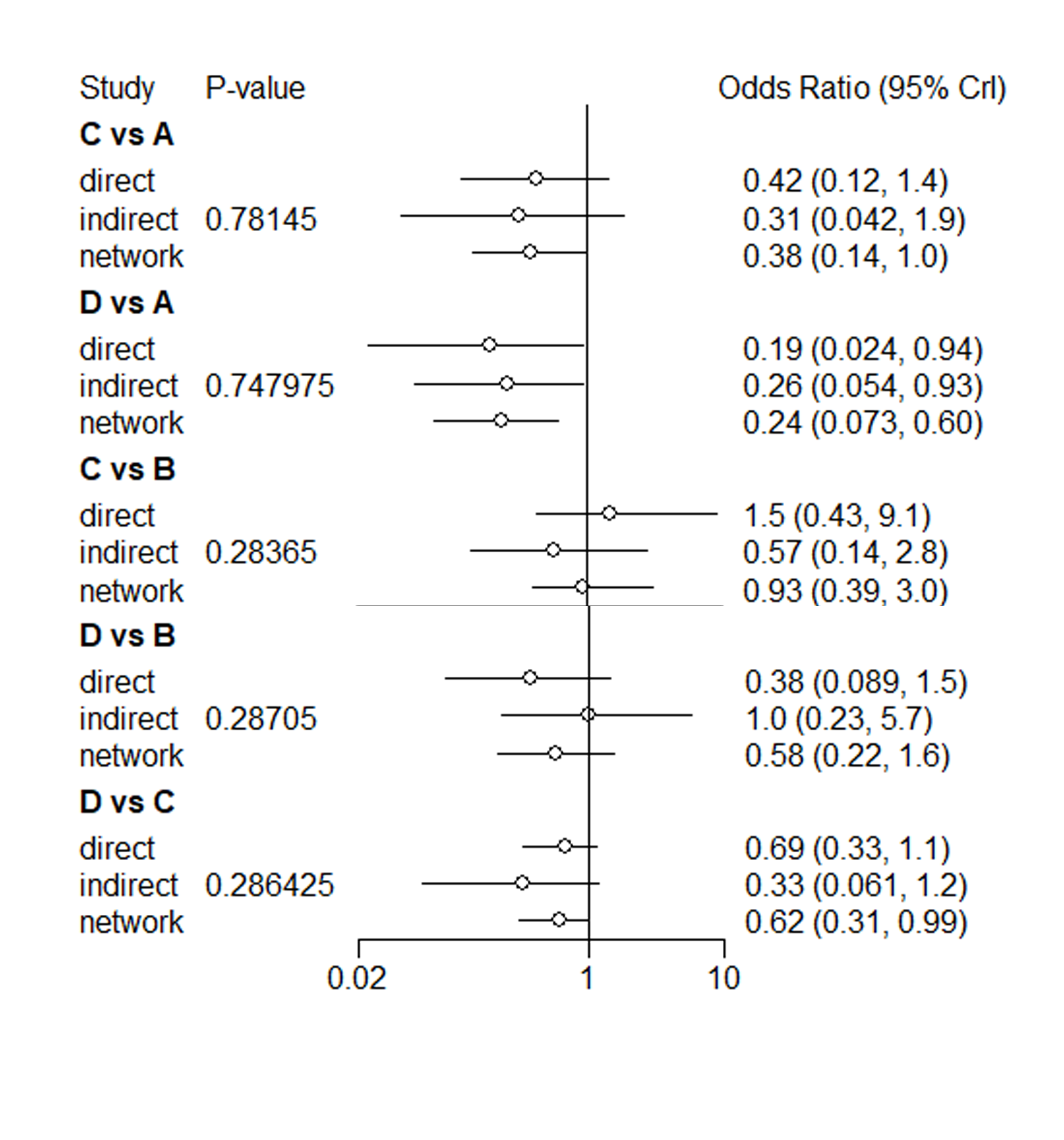

Supplement: Supplementary Figure 1 — Network node-splitting analysis for complete remission. (A) Rituximab (RTX); (B) Tacrolimus (TAC); (C) Mycophenolate mofetil (MMF); (D) Cyclophosphamide (CYC); 95%CrI, 95% credibility interval. [file Image_1.tif]

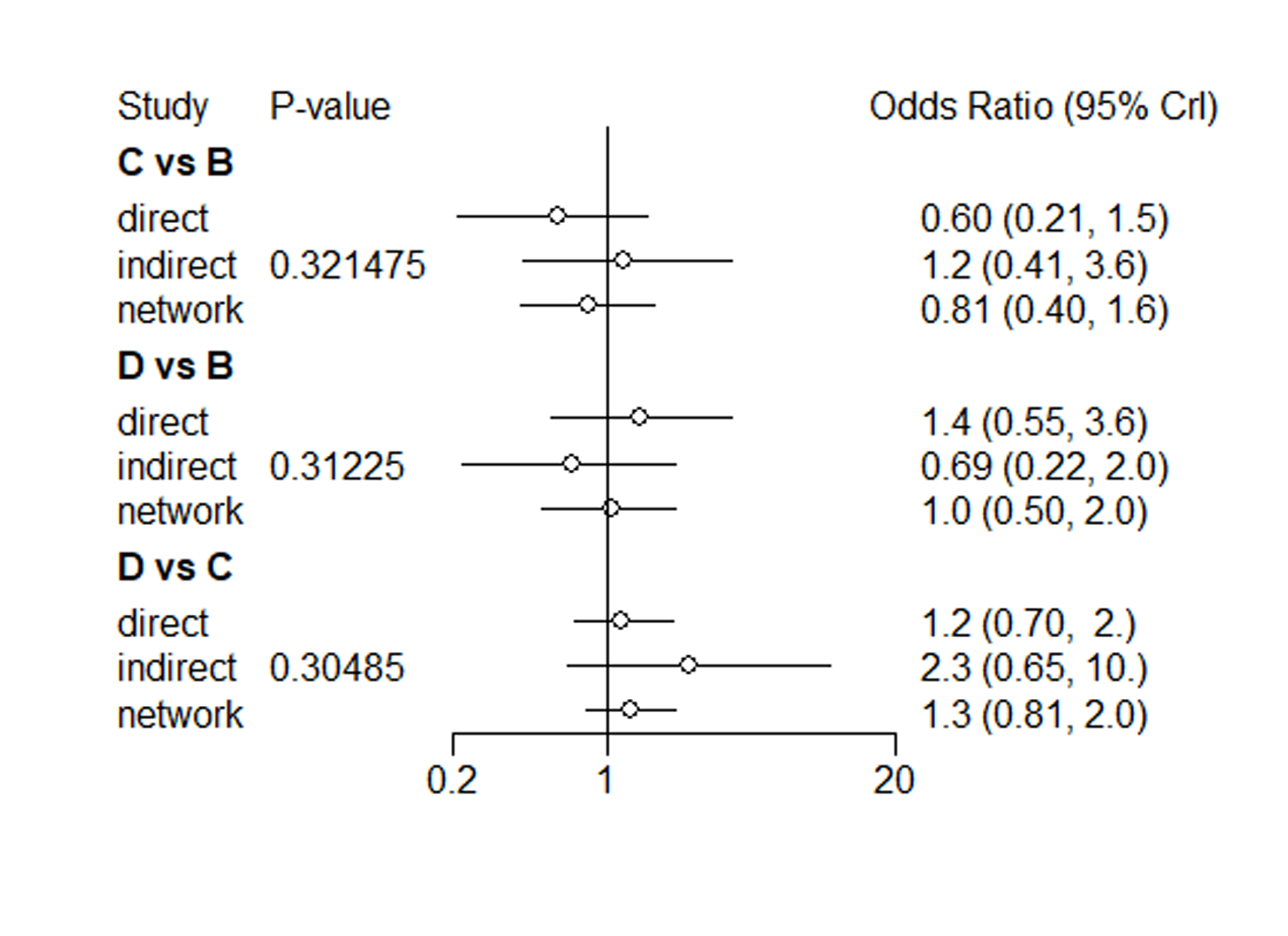

Supplement: Supplementary Figure 2 — Network node-splitting analysis for partial remission. (B) Tacrolimus (TAC); (C) Mycophenolate mofetil (MMF); (D) Cyclophosphamide (CYC); 95%CrI, 95% credibility interval. [file Image_2.tiff]

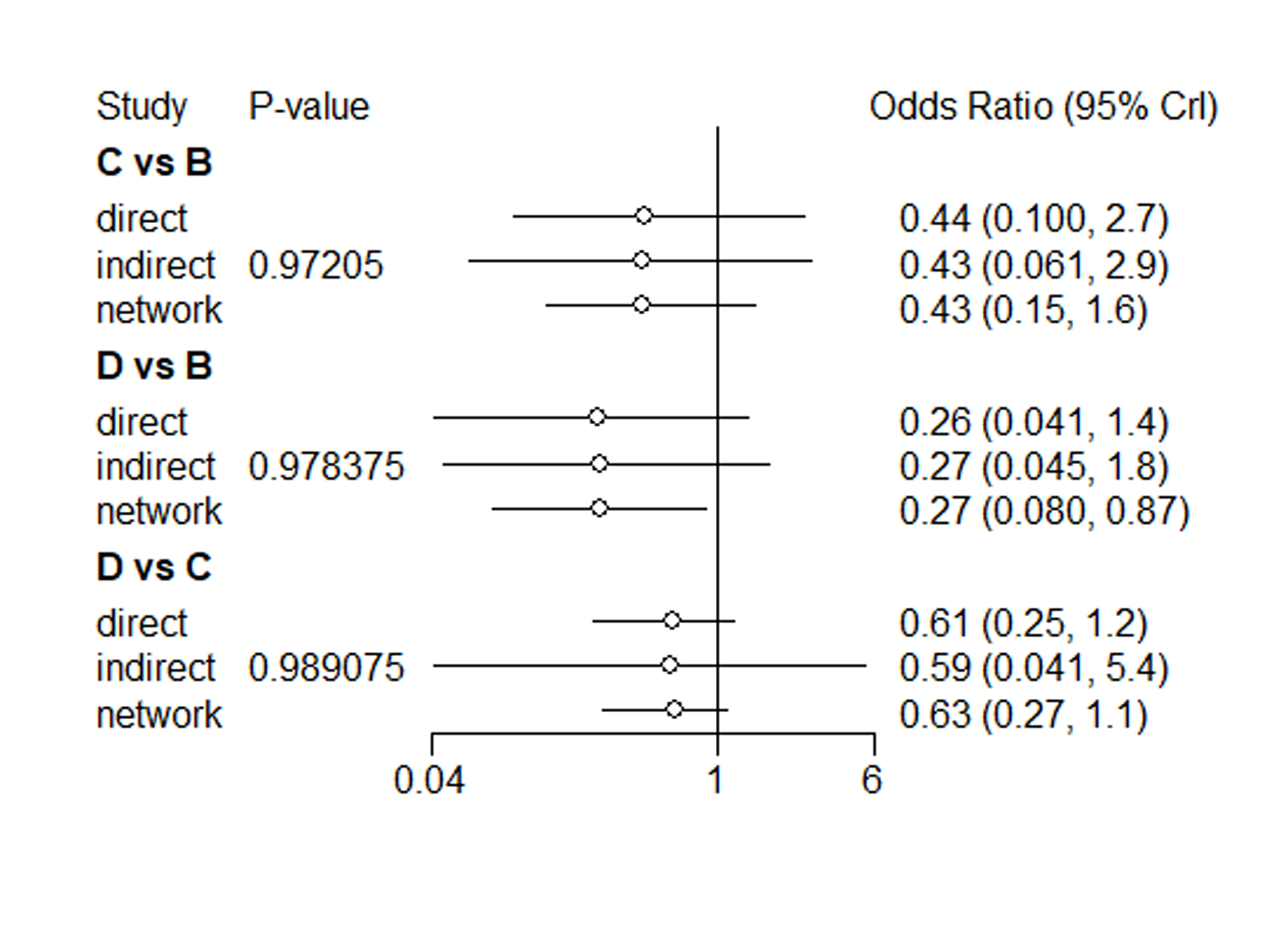

Supplement: Supplementary Figure 3 — Network node-splitting analysis for overall response. (B) Tacrolimus (TAC); (C) Mycophenolate mofetil (MMF); (D) Cyclophosphamide (CYC); 95%CrI, 95% credibility interval. [file Image_3.tiff]

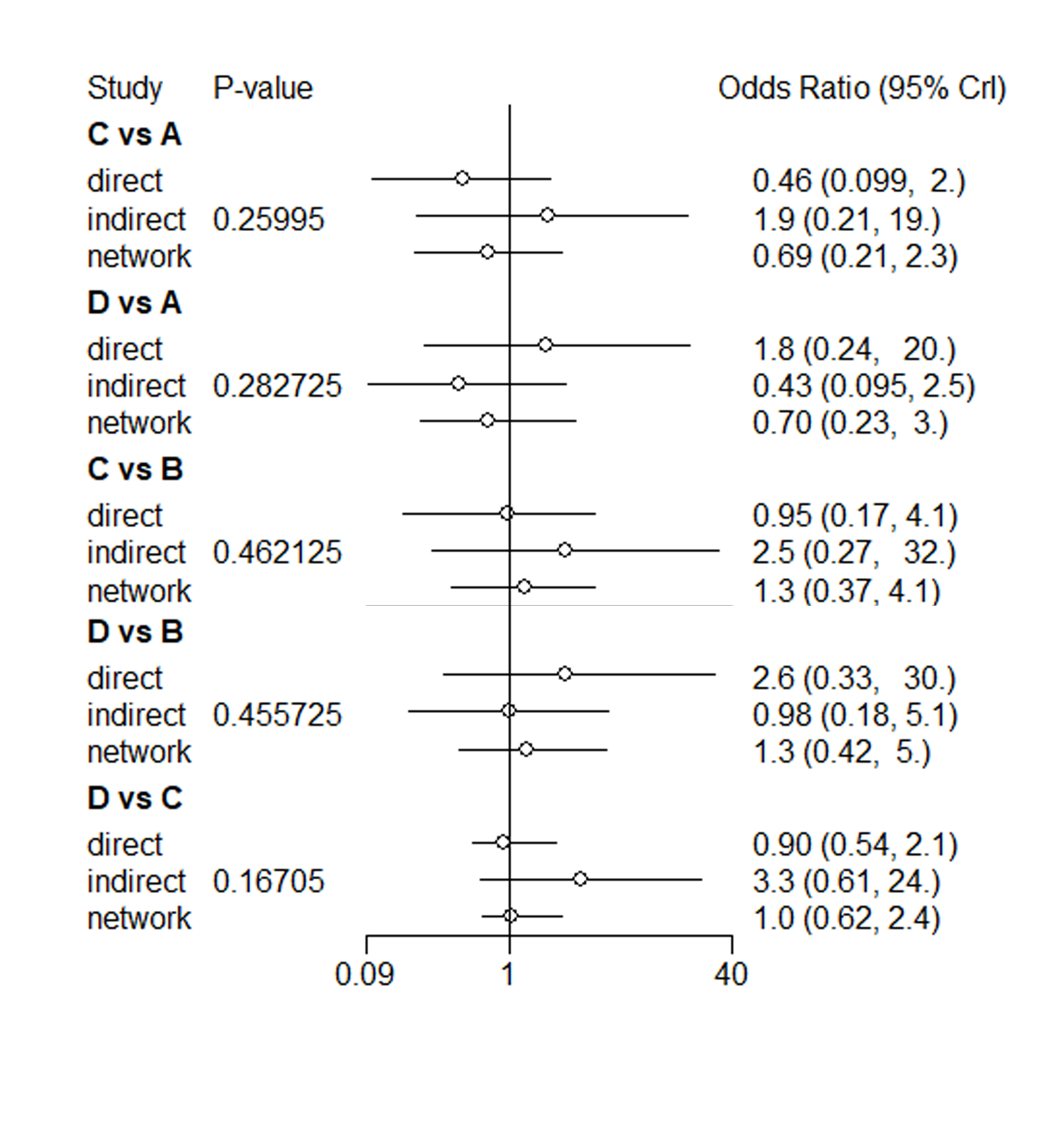

Supplement: Supplementary Figure 4 — Network node-splitting analysis for infection. (A) Rituximab (RTX); (B) Tacrolimus (TAC); (C) Mycophenolate mofetil (MMF); (D) Cyclophosphamide (CYC); 95%CrI, 95% credibility interval. [file Image_4.tif]

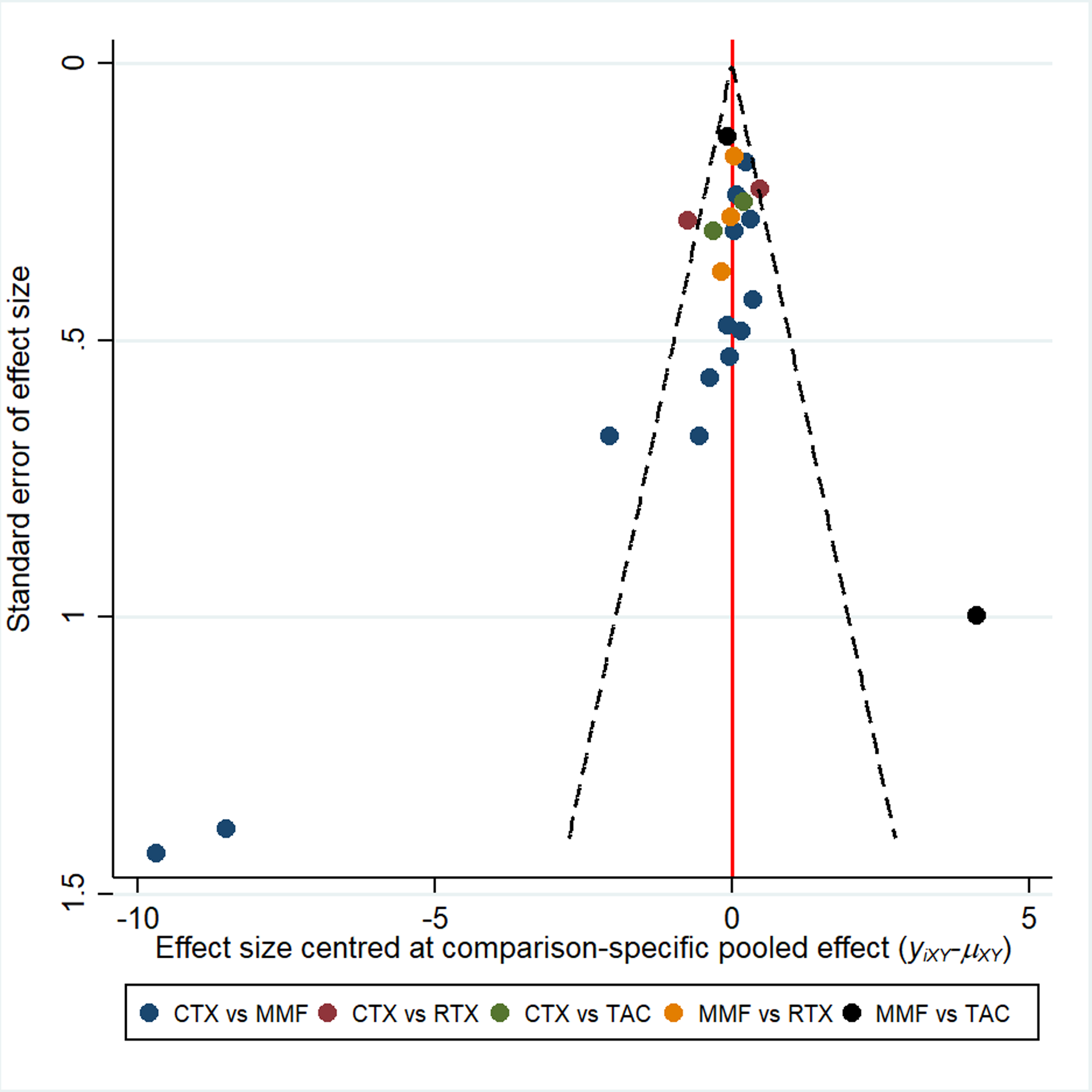

Supplement: Supplementary Figure 5 — Funnel plot for complete remission. RTX, Rituximab; TAC, Tacrolimus; MMF, Mycophenolate mofetil; CYC, Cyclophosphamide. [file Image_5.tif]

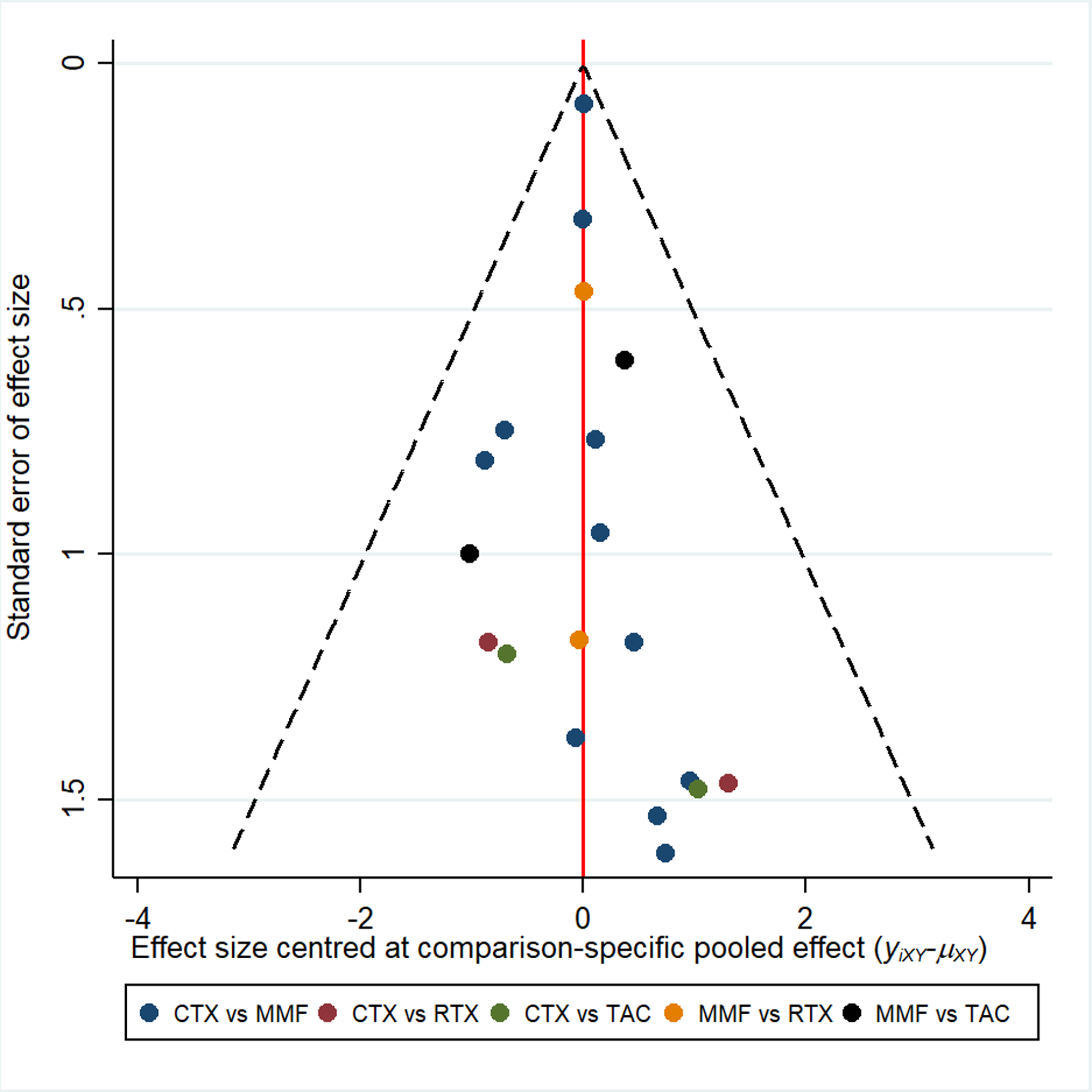

Supplement: Supplementary Figure 6 — Funnel plot for partial remission. RTX, Rituximab; TAC, Tacrolimus; MMF, Mycophenolate mofetil; CYC, Cyclophosphamide. [file Image_6.tif]

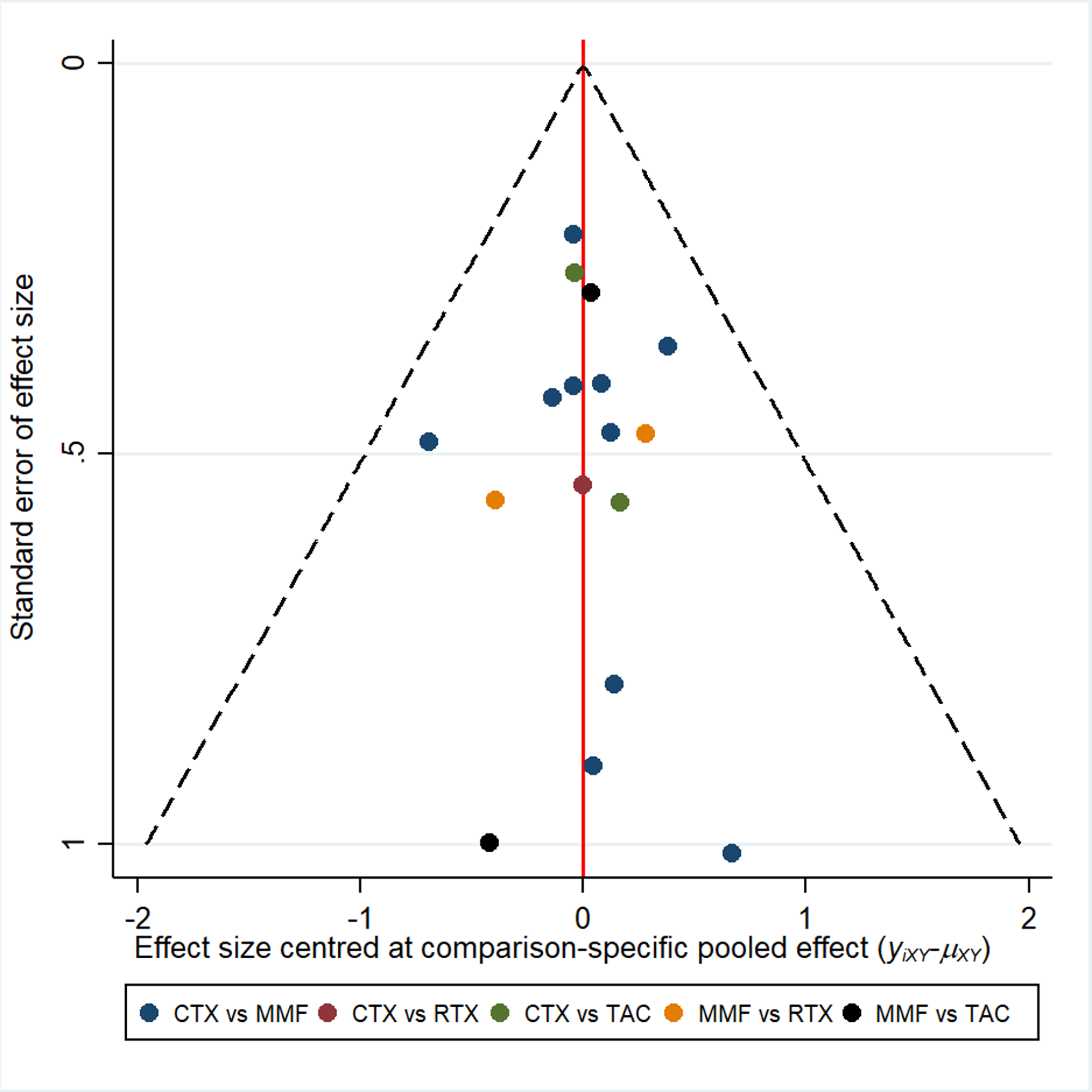

Supplement: Supplementary Figure 7 — Funnel plot for overall response. RTX, Rituximab; TAC, Tacrolimus; MMF, Mycophenolate mofetil; CYC, Cyclophosphamide. [file Image_7.tif]

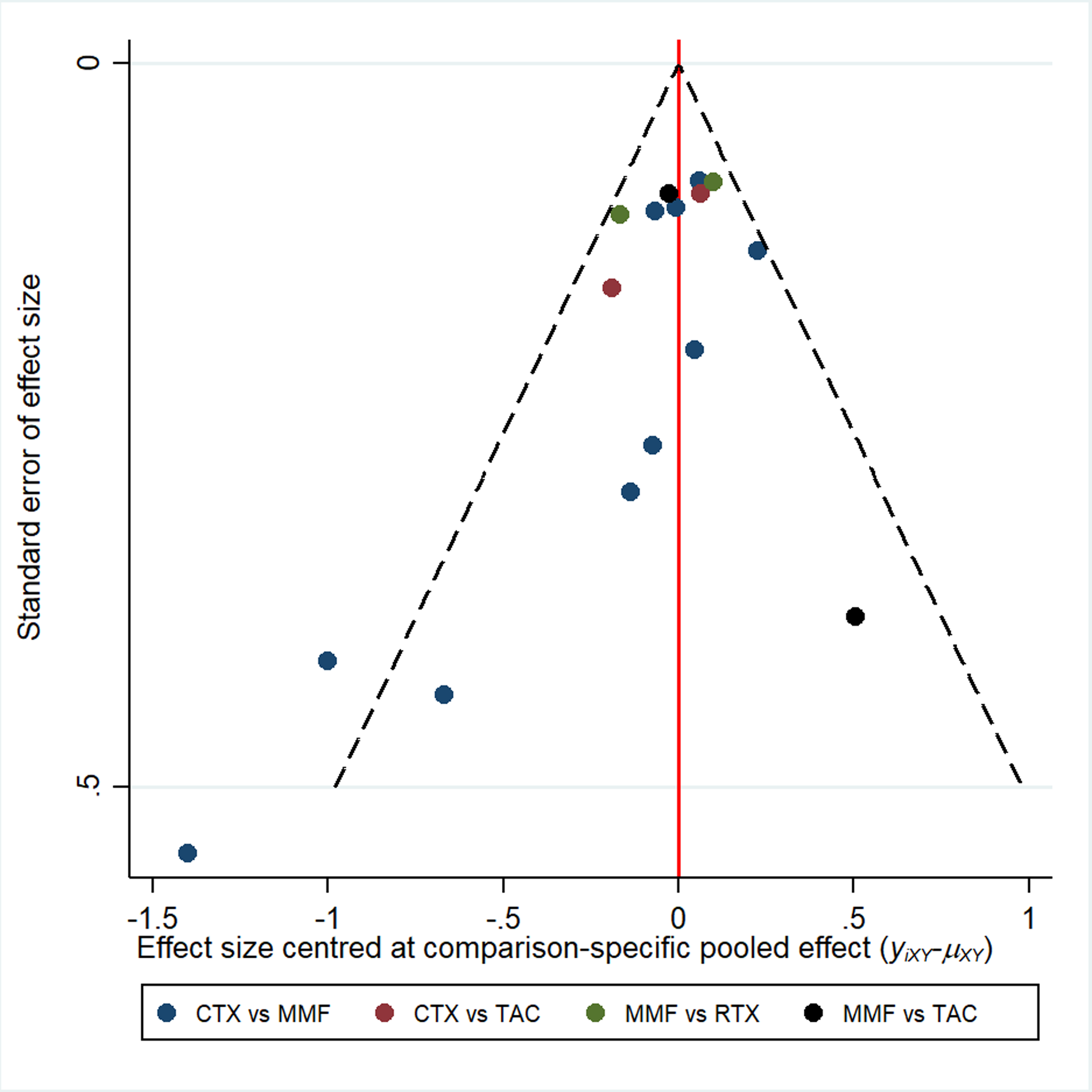

Supplement: Supplementary Figure 8 — Funnel plot for infection. RTX, Rituximab; TAC, Tacrolimus; MMF, Mycophenolate mofetil; CYC, Cyclophosphamide. [file Image_8.tif]
